# Preventive Effects of Long-Term Intake of Plant Oils With Different Linoleic Acid/Alpha-Linolenic Acid Ratios on Acute Colitis Mouse Model

**DOI:** 10.3389/fnut.2022.788775

**Published:** 2022-07-12

**Authors:** Xianshu Wang, Hao Yue, Haonan Zhang, Lei Wan, Shuxia Ji, Chong Geng

**Affiliations:** ^1^Department of Breast and Thyroid Surgery, Shandong Provincial Hospital Affiliated to Shandong First Medical University, Jinan, China; ^2^Shandong Provincial Key Laboratory of Plant Stress Research, College of Life Sciences, Shandong Normal University, Jinan, China; ^3^Department of Endocrine and Metabolic Diseases, Affiliated Hospital of Wei Fang Medical University, Weifang, China; ^4^Shandong Academy of Agricultural Science, Jinan, China

**Keywords:** linoleic acid, alpha-linolenic acid, colitis mouse model, inflammation, gut microbiota, 16S rRNA gene sequencing

## Abstract

**Objective:**

To investigate the preventive effects of plant oils with different linoleic acid/alpha-linolenic acid (LA/ALA) ratios against colitis symptoms, and dysbiosis of gut microbiota in acute colitis mouse model.

**Methods:**

Sixty male C57BL/6 mice were assigned into six groups (*n* = 10): three groups were fed low-fat diets with low, medium, and high LA/ALA ratios; and three groups were fed with high-fat diets with low, medium, and high LA/ALA ratios. After 3 months of diet, the mice were exposed to dextran sodium sulfate solution to induce acute colitis. The severity of colitis was estimated by disease activity index (DAI) and histopathological examination. *16S rRNA* gene sequencing was used for the analysis of gut microbiota.

**Results:**

Plant oils with a lower LA/ALA ratio showed higher alleviating effects on the symptoms of colitis, which were accompanied by the better prebiotic characteristics manifested as effectively inhibiting the abnormal expansion of phylum *Proteobacteria* and genus *Escherichia-Shigella* in the gut microbiota of colitis mouse models.

**Conclusion:**

A potential IBD prevention strategy of reducing the LA/ALA ratio in the daily consumed plant oils was proposed in this study. Furthermore, based on the optimized LA/ALA ratio, this preventive effect might not be weakened by the high intake of plant oils.

## Introduction

As a chronic and relapsing immune-mediated inflammatory disease, the inflammatory bowel disease (IBD) is presented with the symptoms of bodyweight loss, abdominal pain, diarrhea, fatigue, and rectal bleeding (1), and is usually accompanied by an increased risk of colon cancer (2). Due to the complex etiology of IBD, there are still no effective treatment methods for its complete curing. Furthermore, the use of traditional immunotherapy for the IBD treatment, such as azathioprine, might increase the risk of various cancers, including lymphoid tissue cancer, urinary tract cancer, and colon cancer (3, 4). Therefore, since the IBD is becoming a public health concern, investigating its prevention strategies is of great practical significance. Increasing evidence suggests that the dietary and nutritional factors participate in the pathogenesis of IBD (5); the potential of these two key factors as therapeutic options should not be ignored for the development of IBD prevention strategies (6).

As a collective term for the microorganisms, the gut microbiota performs indispensable functions in digestion, absorption, metabolism, immunity, etc. (2, 7). Accumulating studies indicate that the dysbiosis of gut microbiota plays a fundamental role in the pathogenesis of IBD and IBD-associated colon cancer, and has become a potential target for IBD prevention (2). The main manifestation of the dysbiosis of gut microbiota is a reduction in biodiversity (8, 9) as well as an imbalance between the putative protective and harmful species in gut microbiota (1, 10). For example, a reduction in the relative abundance of dominant phyla in the commensal gut microbiota, including *Firmicutes* and *Bacteroidetes*, as well as the abnormal increase in the relative abundance of phylum *Proteobacteria* have been confirmed as a signature of the dysbiosis of gut microbiota in IBD patients (11, 12). Specific opportunistic pathogens, such as *Escherichia coli* (*E. coli*), have been proven to promote the pathogenesis of IBD to a certain extent (13). Therefore, re-establishing the balance of gut microbiota has a very broad prospect for the treatment of IBD (14, 15). As diet is the most direct factor, affecting the composition of gut microbiota, the dietary intervention can also be regarded as a potential IBD treatment strategy, targeting the gut microbiota (16).

Western diet has been identified as a key factor related to the increasing incidences of IBD (5). An obvious characteristic of the western diet is the high-fat diet. Animal experiments confirmed that a high-fat diet could promote intestinal inflammation (17, 18). However, a population survey showed conflicting results, and most of the studies have also proved a positive correlation between a high-fat diet with IBD (19). On the other hand, using two large prospective cohorts of women, Ananthakrishnan et al. also reported that the amount of fat could not influence the risk of IBD (20).

Another major characteristic of the western diet is favoring the production of n-6 polyunsaturated fatty acids (PUFAs), resulting in a great difference in the n-6/n-3 PUFAs ratio as compared to the healthy traditional diets (21). From the perspective of the possible correlation between the unsaturated fatty acid composition and IBD, it was reported that the intake of n-6 PUFAs was positively associated with the IBD risk, whereas the n-3 PUFAs (specifically docosahexaenoic acid) was related to the reduction of IBD risk (22). However, a systematic review, based on 19 identified studies, displayed the lack of the protective effects of n-3 PUFAs against IBD (19). These conflicting results suggest that further investigations of the contributions of n-3 and n-6 PUFAs in the occurrence of IBD are needed.

In this study, we aimed to evaluate the preventive effects of adjusting the ratio of n-6/n-3 PUFAs in the dietary fat for the prevention of IBD. To that end, the preventive effects of the long-term intakes of plant-derived oils with three different precise linoleic acids (LA)/alpha-linolenic (ALA) (LA is an n-6 PUFA, ALA is an n-3 PUFA) ratios were studied in the dextran sodium sulfate (DSS)-induced colitis mouse models. Furthermore, two different levels of fat contents, including high- and low-fat contents, were set in each set of LA/ALA ratios. This study could fill two obvious research gaps in the previous results of population surveys and animal experiments: (1) The most recent studies, especially in a population survey, focused on the n-3 PUFAs, especially DHA (docosahexaenoic acid) and EPA (eicosatetraenoic acid), which originate from fish oils (23); (2) It was worth noting that in the animal colitis model studies, the saturated fatty acids-rich animal fat, lard, was generally used in a high-fat diet, while there are only few animal studies based on the plant-derived oil, which are rich in the unsaturated fatty acids (17, 18, 24). Since the consumption of plant-derived oils is essential in daily diet, this study is of great value to provide easy-to-implement diet guidance for preventing or alleviating the clinical symptoms of IBD and the dysbiosis of gut microbiota associated with IBD.

## Materials and Methods

### Animals

**FIGURE 1:**
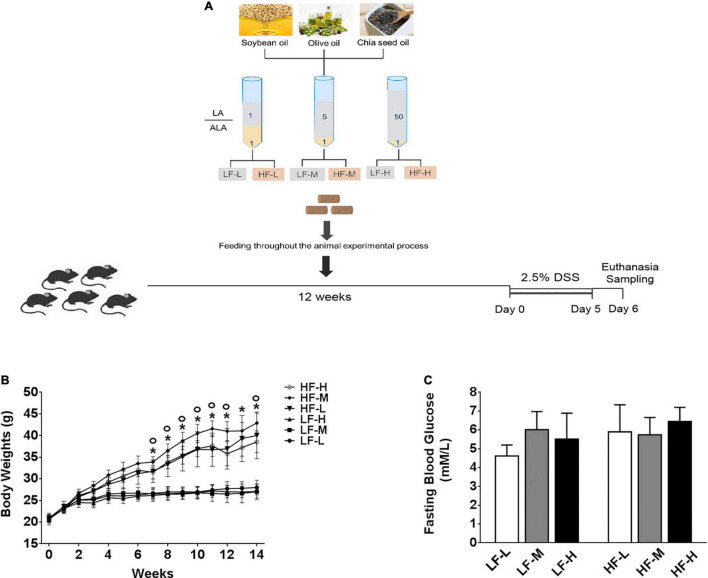
**(A)** Illustration of experimental design and basic indices of mice during 3 months on different diets. **(B)** Curve of changes in body weights. **(C)** Fasting blood glucose levels after 3 months feeding on different diets. Asterisk indicates a significant difference between the high-fat diet groups and low-fat diets (LF-L + LF-M + LF-H vs. HF-L + HF-M + HF-H) and circle indicates a significant difference between the HF-M and other high fat diet groups.

All the animal protocols were performed with the approval of the Animal Care and Use Ethics Committee of the Shandong Provincial Hospital Affiliated to Shandong First Medical University (Shandong, China) under the approval number NO. 2021-482. Sixty mice (male, C57BL/6, 6 weeks old) were purchased from the GemPharmatech LLC. (Jiangsu, China), acclimatized for 2 weeks, and then assigned into six weight-matching groups (*n* = 10). Each group of mice was fed with one of the six designed animal diets. Mice were housed in a facility with a controlled environment (12/12 h daylight cycle) and access to food and water *ad libitum*. The body weights and diet consumption of mice were monitored and recorded weekly. The fasting blood glucose levels before exposure to DSS solution were measured using a glucometer (Roche Diagnostics, Manheim, Germany) by collecting blood from their tail vein after an overnight 14 h fasting. The details of the experimental diets are provided below. After 12 weeks on specific diets, the mice were induced to develop acute colitis by intaking 2.5% DSS (MP Biomedicals, United States) solution with molecular weight, ranging from 36–50 kDa, for 5 consecutive days. The animal experiment design is illustrated in Figure 1A.

### Diets

The soybean, olive, and chia seed oils were mixed in different proportions to prepare the following three kinds of mixed oils with different LA/ALA ratios, including 1:1, 5:1, and 50:1, respectively. Six experimental diets, containing low (4.27% plant oils, 10% kcal fat) or high (23.60% plant oils, 45% kcal fat) levels of mixed plant oils, were designed as follows. The 3 low-fat experimental diets, containing plant oils with low (1:1), medium (5:1), and high (50:1) LA/ALA ratios, were named LF-L, LF-M, and LF-H, respectively, while the 3 high-fat experimental diets, containing plant oils with low (1:1), medium (5:1) and high (50:1) LA/ALA ratios, were named HF-L, HF-M, and HF-H, respectively.

The animal diet ingredients and fatty acids compositions of mixed plant oils are provided in Supplementary Tables 1, 2. All the diets were customized by Huafukang Bioscience Co. (Beijing, China).

### Evaluation of Disease Severity Using Disease Activity Index

As previously described (25), the progression of colitis was evaluated by calculating the DAI scores daily. The DAI scores were calculated by combining the measured scores of three categories on a scale from 0 to 10, including bodyweight (0–4), stool consistency by visual inspection (0–3), and status of the rectal bleeding. The detailed information on each indicator is provided in Supplementary Table 3.

### Animal Tissue Samples Collection

The procedures for tissue collection have been described previously (25). In brief, after the induction of intestinal colitis, the mice were sacrificed by cervical dislocation under isoflurane-induced anesthesia. Then, the chest of each mouse was opened and their intestines were separated. The cecum contents were snap-frozen for microbial analysis. After isolating from the chest, their colon length and spleen weight were recorded.

### Histopathological Analysis of Colon Tissue

A 2-cm long colon segment was used for the histopathological analysis. For this purpose, the colon segments were opened longitudinally, embedded in paraffin after formalin fixation, cut into 5-μm-thick sections, treated with xylene substitute and gradient ethanol solutions, stained with hematoxylin and eosin in sequence, and finally examined under an optical microscope. For each section, eight microscopic fields were randomly selected for the evaluation of histopathological scores. The severity of colonic lesions caused by the disease was reflected by the sum of three indicators, including colonic epithelial damage (0–4), inflammation severity (0–3), and inflammatory infiltrate (0–3) as described previously (25).

### Analysis of the Composition of Gut Microbiota Using 16S Ribosomal RNA Gene Sequencing

The quantitative analysis of bacterial species in gut microbiota was performed according to the protocol described in a previous study (26). Total bacterial DNA for the gut microbial analysis was extracted from frozen cecum contents using a Tiangen Stool DNA Extraction Kit (Tiangen Biotech Co., Ltd., Beijing, China). The V3-V4 hyper-variable region of the *16S rRNA* gene was amplified using a limited cycle PCR. The amplified products were purified by the magnetic bead method. The DNA libraries were constructed, following the manufacturer’s instructions, and then a paired-end sequencing was run on an Illumina HiSeq-2000 platform (Illumina, Inc., CA, United States). The result in *16S rRNA* gene sequencing raw data was processed using the Quantitative Insights Into Microbial Ecology (QIIME) software package (V.1.9.1) (27, 28). The paired-end reads were de-multiplexed and joined using FLASH v1.2.7 and then quality filtered. The resulting tags were assigned to the Operational Taxonomic Units (OTUs) with a 97% similarity cut-off. The identified taxonomy was then aligned against the Silva reference database (version 128)^1^ (28). The raw sequencing data were deposited in the NCBI database under the BioProject accession number PRJNA820301.

### Statistical Analysis

The datasets were analyzed using two-way ANOVA, followed by Tukey *post hoc* tests (the two analyses factors were LA/ALA ratio and fat concentration in diets), and the correlations between different groups were analyzed with Pearson’s correlation using GraphPad Prism version 8.00 (GraphPad Software, Inc., CA, United States). A *P-*value lower than 0.05 was considered statistically significant.

## Results

### Effects of Different Linoleic Acid/Alpha-Linolenic Acid Ratios on the Basic Parameters in Mice Before Dextran Sodium Sulfate-Induction

As shown in Figure 1B, during the 12-weeks (3 months) period on different diets, the weight gain of the three high-fat diets (HF-L, HF-M, HF-L) feeding mice groups was significantly higher than that of low-fat diets feeding mice (LF-L, LF-M, LF-H) groups from the fifth week onward. By comparing the bodyweight at the same level of fat concentration, the difference between the mice groups fed with low-fat diets was not significant. The comparisons of high-fat diet groups showed that the growth rate of the bodyweight of HF-M group mice was significantly higher than that of the HF-L and HF-H groups from the eighth week onward. After 3 months of feeding, the bodyweight of HF-M group mice was significantly higher than that of HF-H and HF-L group mice. Furthermore, as shown in Figure 1C, there was no significant difference in the fasting blood glucose levels among the mice fed with different diets.

### Physiological Parameters of Dextran Sodium Sulfate-Induced Colitis Mouse Models

**FIGURE 2:**
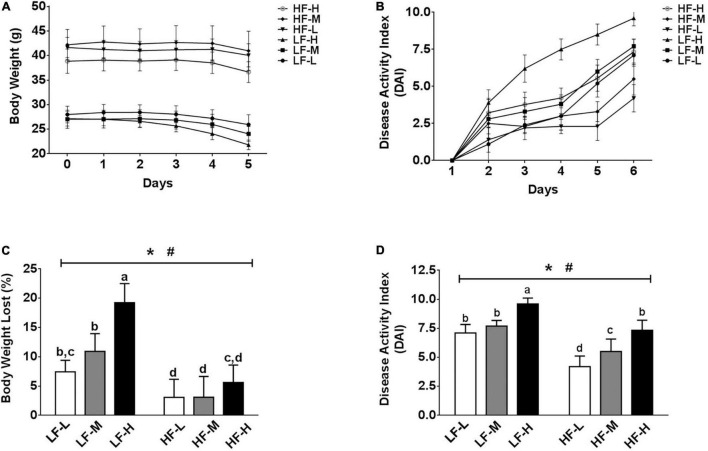
Body physiological conditions and DAI scores. **(A)** Change curves of body weight and **(B)** DAI scores during DSS-induction. Comparison of **(C)** body weights and **(D)** DAI scores at the end of DSS-induction. Bars with the same letter indicate a non-significant difference (*P* > 0.05). Asterisk indicates a significant difference between the high fat diet groups and low fat diet groups; ^#^indicates a significant difference between the mice from groups fed with the diets of different LA/ALA ratios.

Regardless of the diets, all mice exposed to DSS showed certain symptoms of colitis, but the severity of the symptoms of colitis was different between the groups. As shown in Figure 2A, irrespective of the fat concentration, the bodyweight of the low LA/ALA ratio groups was the most stable, followed by the medium LA/ALA ratio groups, while that of the high LA/ALA ratio groups showed obvious changes. The measurement of DAI scores during the DSS induction showed a similar trend. Moreover, the disease progression in the LH-H group was the fastest among all of the six groups (Figure 2B).

As the statistical results showed in Figures 2C,D, both the fat contents and ratios of LA/ALA were the key factors, affecting the progression of colitis in mice. After induction of colitis, two important indicators used to characterize the severity of colitis, including bodyweight loss rate and DAI score, were significantly higher in the low-fat diets groups as compared to those of the high-fat diets groups. Considering the same fat concentration level, the bodyweight loss rates and DAI scores were significantly higher in the LH-H group than those of the LH-L and LH-M groups; a similar trend also occurred in high-fat groups, suggesting that the increase in LA/ALA ratios in the diet significantly aggravated the colitis symptoms in mice.

### Histopathological Indices of Colon Tissue in Dextran Sodium Sulfate-Induced Colitis Mice

**FIGURE 3:**
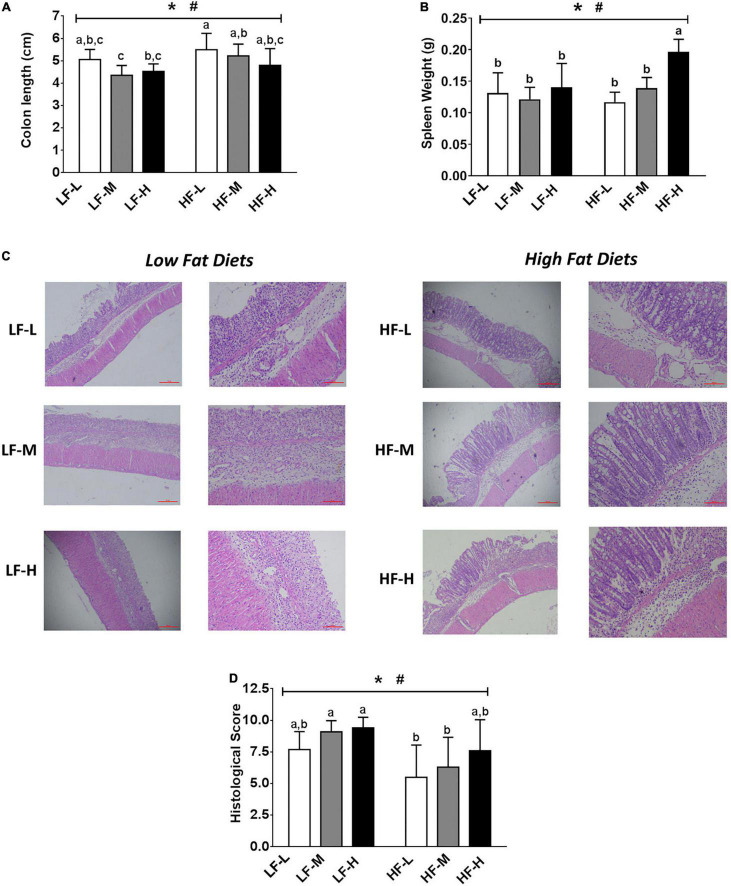
**(A)** Colon lengths of the mice. **(B)** Spleen weights of mice. **(C)** Histopathological examination and **(D)** evaluation of histopathological severity scores of the colon tissue. Bars with the same letter indicate a non-significant difference (P > 0.05). Asterisk indicates a significant difference between the high fat diet groups and low fat diet groups; ^#^indicates a significant difference between the mice from groups fed with the diets of different LA/ALA ratios.

The shortened colons and enlarged spleens were the important symptoms of colitis (Figures 3A,B). As compared to the low-fat diets groups, the high-fat diets significantly alleviated the shortening of colon length induced by colitis (*P* = 0.0016). For the spleen weight, the HF-H group had the most obviously enlarged spleen among all of the dietary groups, indicating that the high-fat diet with a high LA/ALA concentration showed obvious pro-inflammatory effects.

The colonic inflammation and epithelial injury were observed in all the DSS-induced mice (Figure 3C). The summarized histopathological scores (Figure 3D) indicated that the intestinal damage of mice in high-fat diet groups was significantly relieved as compared to that of the low-fat diet groups (*P* = 0.0016). Consistent with DAI scores, the severity of intestinal damage aggravated with the increase in LA/ALA concentrations in diets; this trend was particularly evident in the high-fat diets groups.

In general, the increase in the concentration of ALA in diets exerted positive effects in relieving colitis symptoms. The colitis symptoms in the mice fed with high-fat diets were significantly relieved when compared to those fed with low-fat diets.

### Disturbance of Bacterial Diversity and Bacterial Communities in Gut Microbiota

**FIGURE 4:**
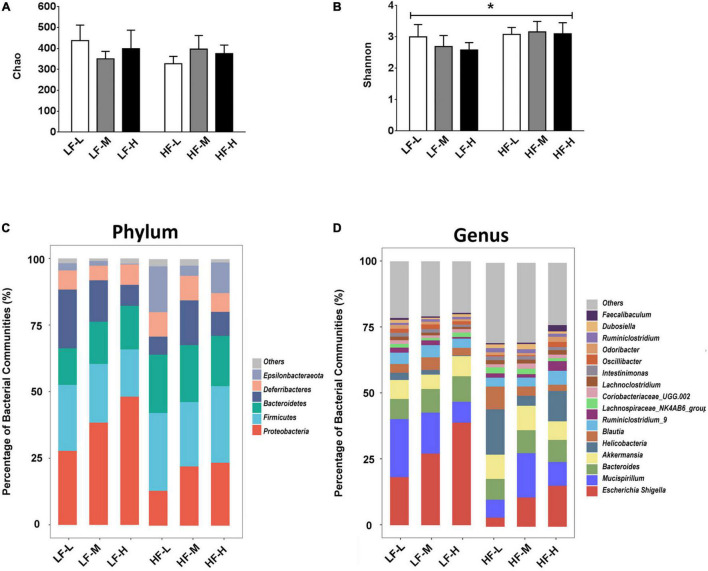
**(A)** Bacterial richness of gut microbiota evaluated by Chao index. **(B)** Bacterial diversity of gut microbiota estimated by Shannon index. **(C)** Compositions of gut microbiota in different groups at phylum level, and **(D)** compositions of gut microbiota in different groups at genus level. Asterisk indicates a significant difference between the high fat diet groups and low fat diet groups.

In order to investigate the effects of different diet factors on the richness and diversity of gut microbiota, the Chao and Shannon indices for the richness and diversity of species, respectively, were calculated and presented in Figures 4A,B. The evaluation results of the Chao index showed that the different types of diets in this study did not change the species richness, irrespective of the fat or LA/ALA concentrations in the animal diets. However, according to Shannon index, the mice fed with high-fat diets showed a significant increase in their bacterial diversity than those fed with low-fat diets (*P* = 0.0076). No significant variations in the species diversity were indicated by the Shannon diversity index between the groups fed with different ratios of LA/ALA diets.

As illustrated in Figure 4, different diets induced obvious differences in the composition of gut microbiota. At the phylum level (Figure 4C), *Proteobacteria* represented the most abundant bacterial phylum in the low-fat diet groups (LF-L: 27.77%; LF-M: 28.37%; LF-H: 48.11%), especially in the LF-H group, their abundance was almost half of the gut microbiota. The most dominant bacterial phylum was *Bacteroidetes* in the high-fat diet groups (HF-L: 29.23%; HF-M: 24.18%; and HF-L: 28.76). At the genus level (Figure 4D), *Escherichia-Shigella* was the most abundant genus in all the dietary groups (LF-L: 18.20%; LF-M: 27.15%; LF-H: 38.86%; HF-M: 11.05%; HF-H: 15.49) except the HF-L group.

### Analysis of Main Bacterial Communities in Dextran Sodium Sulfate-Induced Colitis Mouse Models

**FIGURE 5:**
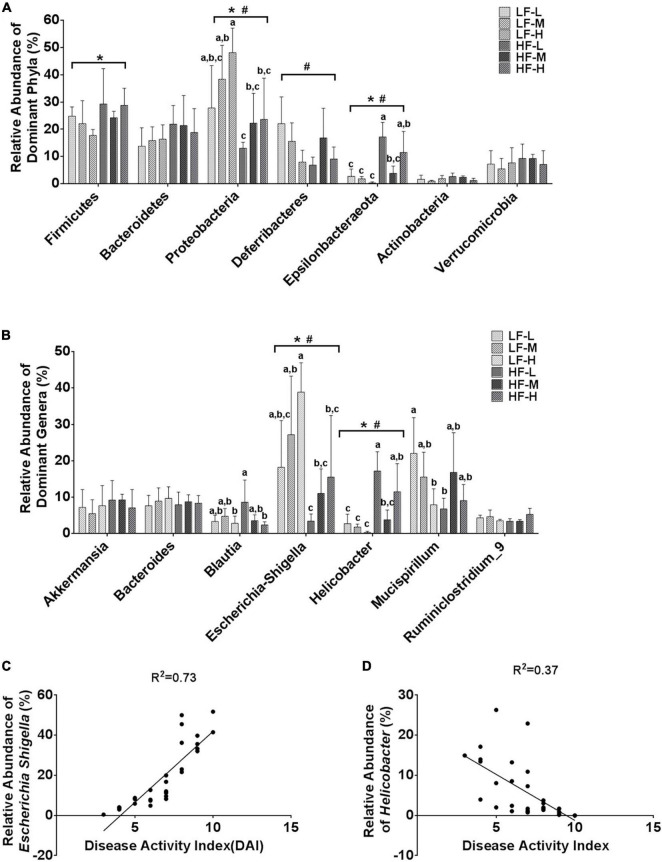
Statistical comparisons of the relative abundances of the **(A)** top 7 main bacterial phyla and **(B)** top 7 main bacterial genera in the gut microbiota of mice from the groups fed with different diets. **(C)** Correlations between the relative abundance of genus *Escherichia Shigella* and DAI scores. **(D)** Correlations between the relative abundance of genus *Helicobacter* and DAI scores. Bars with the same letter indicate a non-significant difference (*P* > 0.05). Asterisk indicates a significant difference between the high fat diet groups and low fat diet groups; ^#^indicates a significant difference between the mice from groups fed with the diets of different LA/ALA ratios.

In order to further characterize the prebiotic effects of different LA/ALA ratios on the homeostasis of gut microbiota, the relative abundances of the seven main phyla and the seven most abundant genera were compared. Among the seven phyla, *Proteobacteria* (*P* = 0.024), *Deferribactere* (*P* = 0.0011), and *Epsilonbacteraeota* (*P* = 0.0028) were significantly affected by the administration of plant oils with different LA/ALA ratios (Figure 5A). On the other hand, the phyla *Firmicutes* (*P* = 0.033), *Proteobacteria* (*P* = 0.003), and *Epsilonbacteraeota* (*P* < 0.0001) were significantly affected by the fat concentrations in the diet, as shown by the results of two-way ANOVA analysis (Figure 5A). The relative abundances of *Proteobacteria* and *Epsilonbacteraeota* in gut microbiota were influenced by both the LA/ALA ratios and fat concentrations in diets. A trend that could be easily noticed was the increase in the abundance of the *Proteobacteria* with the increase in the ratio of LA/ALA in the diet (Figure 5A). In addition, the relative abundance of phylum *Proteobacteria* in gut microbiota was significantly higher in DSS-induced mice fed with low-fat diets as compared to those fed with high-fat diets (Figure 5A). In the gut microbiota of mice fed with low-fat diets, the abundance of phylum *Epsilonbacteraeota* decreased with the increase in LA/ALA ratio in diets, although the difference was not significant. However, in the gut microbiota of mice fed with high-fat diets, the relative abundance of *Epsilonbacteraeota* was the lowest in the medium LA/ALA ratio diet group.

According to the statistical analysis results at the genus level (Figure 5B), the relative abundances of *Escherichia-Shigella* and *Helicobacter* in gut microbiota were significantly affected by both the LA/ALA ratios and fat concentrations in diets. *Escherichia-Shigella* was significantly more abundant in the gut microbiota of mice fed with low-fat diets as compared to those fed with high-fat diets. Moreover, irrespective of the fat’s concentration, the relative abundance of *Escherichia-Shigella* was proportional to the LA/ALA ratio (LF-H > LF-M > LF-L; HF-H > HF-M > HF-L). The relative abundance of *Helicobacter* was significantly higher in high-fat diet groups as compared to that in the low-fat diet groups. In the gut microbiota of mice fed with low-fat diets, the abundance of genus *Helicobacter* decreased with the increase in LA/ALA ratio in diets, although the differences among groups were not significant. In the gut microbiota of mice fed with high-fat diets, the relative abundance of *Helicobacter* was the lowest in the medium LA/ALA ratio diet group.

According to taxonomic classification, *Escherichia-Shigella* and *Helicobacter* belong to phyla *Proteobacteria* and *Epsilonbacteraeota*, respectively. Therefore, not surprisingly, the alteration in the relative abundances of *Escherichia-Shigella* and *Helicobacter* showed similar trends to that of phyla *Proteobacteria* and *Epsilonbacteraeota*, respectively. The Pearson’s correlation analysis (Figures 5C,D) revealed a strong positive relationship between the relative abundance of *Escherichia-Shigella* in gut microbiota and DAI score of colitis (*R*^2^ = 0.74; *P* < 0.0001), as well as a weak association between the relative abundance of *Helicobacter* and DAI score of colitis (*R*^2^ = 0.37; *P* = 0.0004) in mouse models.

## Discussion

As stated earlier, the IBD not only seriously endangers human health but is also characterized as a refractory and relapsing disease (29). Therefore, effective treatment strategies for IBD, especially using dietary interventions with low cost and no side effects, are needed to be developed (30). As compared to the studies, concerning the effects of bioactive nutrients, such as multiple sources of polyphenols (31–33), the studies, investigating the relationship between macronutrients in a daily diet and IBD, are relatively limited. Lipid is an essential macronutrient for humans and animals. The widely consumed plant-derived oil is one of the major sources of unsaturated fatty acids, including two kinds of essential fatty acids for the human body, n-3-PUFA, and n-6-PUFA (30, 34).

The current study investigated the effects of the ratio of LA (n-6 PUFA) and ALA (n-3 PUFA) in plant-derived oil and the amount of administered-plant-derived oil with different LA/ALA ratios on the progression of colitis in mice in order to guide the development of plant-derived oil-based treatment strategy for IBD. In order to be closer to reality, a long-term experimental scheme was adopted, which used different LA/ALA ratios and fat concentrations (3 sets of LA/ALA ratios, 2 fat concentrations) before DSS induction. The current study indicated that, at the same fat concentration level, the severity of colitis symptoms was relieved with the decrease in the ratios of LA/ALA. This finding was not unexpected as there are population surveys, reporting that the intake of n-3 PUFA was negatively correlated with the risk of IBD (22, 35). It has been proved that dietary supplementation with food rich in n-3 PUFA can effectively increase the circulating n-3 PUFA level (36). Diwakar et al. reported that the higher ratios of n-6 to n-3 fatty acids were found in the rectal mucosa of the patients with IBD-related joint pain as compared to the controls (37). Moreover, administration of seal oil with high n-3 PUFA content normalized the n-6 to n-3 fatty acids ratio in rectal mucosa and improved the health-related quality of life of the patients with IBD-related joint pain (37, 38). However, it was worth noticing that the studies based on population surveys mainly focused on n-3 PUFA (DHA and EPA), which are present in fish oil, rather than n-3 PUFA from plant oil. Another animal study, focusing on the relationship between different n-6/n-3 PUFA ratios and IBD, also used fish oil as the source of n-3 PUFA rather than plant oil, similar to the population studies (39). This study used plant oils in animal models, thereby fulfilling this gap.

Another concern of this study was the effects of different contents of plant oils (with different LA/ALA ratios) in diets on relieving the symptoms of colitis. The lopsided conclusion of animal studies was that a high-fat diet could promote the development of colitis (24, 40, 41). However, the previous studies used lard for animal diets as the primary fat source. This study showed that the high content of fats (account for 45% of total calories) from plant oils might not prompt the development of colitis. Therefore, it was speculated that if the compositions of fatty acids are healthy enough, even its high intake might not increase the risk of IBD in daily life.

Considering the crucial function of gut microbiota in the initiation and development of IBD (1) and its close relationship with food components (7), in the current study, the gut microbiota in mice was analyzed in order to elucidate the underlying mechanisms, causing significant differences in the severity of colitis symptoms among the different mice groups fed with different diets. The unusual increase in the abundance of phylum *Proteobacteria* has been frequently considered a “microbial signature” for the dysbiosis of gut microbiota (13, 42, 43). It was noticed that the mice fed with plant oils with higher LA/ALA ratios presented more severe characteristics of colitis and a higher abundance of *Proteobacteria* in gut microbiota. This suggested that the higher LA/ALA ratios in plant oil were less conducive to restoring the hemostasis of gut microbiota from the colitis-induced changes in gut microbiota in a mouse model. This study also showed that the gut microbiota of mice fed with high-fat diets showed a higher Shannon index as compared to that of the mice fed with low-fat diets. Although it has been reported that the decreased bacterial richness can be considered a hallmark of the effects of a high-fat diet on the composition of gut microbiota in both humans and mice (44), more recent research conducted a study on the DSS-induced colitis mouse model (same as this study) and demonstrated a higher α-diversity (Shannon index) in the gut microbiota of mice fed with high-fat diet as compared to those fed with a low-fat diet (45). These seemingly contradictory conclusions suggested that the relationship between dietary factors and gut microbiota was highly complex and changeable, thereby requiring further investigations.

A striking prebiotic feature of the lower LA/ALA ratio in plant oil was its significant inhibitory effect on the increasing abundance of *Escherichia-Shigella* in the gut microbiota of DSS-induced acute colitis mouse models. The genus *Escherichia-Shigella* contains several conditioned pathogens, such as *E. coli*, which is one of the most common bacteria implicated in the pathogenesis of IBD (13). The abnormal expansion of genus *Escherichia-Shigella* in gut microbiota has been proved to possibly contribute to the etiology of IBD (13, 46). The strong positive correlation between the relative abundance of *Escherichia-Shigella* and the severity of colitis symptoms (DAI scores) suggested that the alteration of the gut microbiota might be an important factor in explaining the effects of LA/ALA ratios on the severity of colitis symptoms. Furthermore, the prebiotic effect of plant oils with lower LA/ALA ratios on the inhibition of *Escherichia-Shigella* was enhanced rather than weakened when the contents of plant oils in the diet of mice increased to high-fat level.

## Conclusion

There are contradictory conclusions regarding the contribution of n-6 and n-3 PUFAs to the occurrence of IBD, suggesting that there is still a need for reliable studies. In the current study, the evidence, based on animal models, showed that the adjustment of the LA/ALA ratio of edible plant oil in daily diet has a great potential for the prevention of IBD, which might be due to its prebiotic characteristics. Furthermore, on the premise that the composition of fatty acids is reasonable enough, the high intake of vegetable oil might not increase the risk of colitis.

## Data Availability Statement

The original contributions presented in this study are included in the article/Supplementary Material, further inquiries can be directed to the corresponding author.

## Ethics Statement

The animal study was reviewed and approved by the Animal Care and Use Ethics Committee of the Shandong Provincial Hospital Affiliated to Shandong First Medical University (Shandong, China).

## Author Contributions

CG, XW, and HY contributed to the conception and design of the study. XW, HY, HZ, LW, and SJ performed the experiments and analyzed the data. CG and XW wrote the manuscript. All authors contributed to the article and approved the submitted version.

## Conflict of Interest

The authors declare that the research was conducted in the absence of any commercial or financial relationships that could be construed as a potential conflict of interest.

## Publisher’s Note

All claims expressed in this article are solely those of the authors and do not necessarily represent those of their affiliated organizations, or those of the publisher, the editors and the reviewers. Any product that may be evaluated in this article, or claim that may be made by its manufacturer, is not guaranteed or endorsed by the publisher.

## Footnotes

1
http://www.arb-silva.de
