# Supplementary material for: Preventive Effects of Long-Term Intake of Plant Oils With Different Linoleic Acid/Alpha-Linolenic Acid Ratios on Acute Colitis Mouse Model
Source: Front Nutr. 2022 Jul 12;9:788775. doi: 10.3389/fnut.2022.788775 (PMC9315388; doi:10.3389/fnut.2022.788775)
Supplement: Supplementary file 1 [file Data_Sheet_1.PDF]

Preventive Effects of Long-term intake of Plant Oils with Different Linoleic Acid/Alpha-Linolenic Acid Ratios on Acute Colitis Mouse Model

Xianshu Wang[2,4†], Hao Yue[2†], Haonan Zhang[2,4], Lei Wan[3], Shuxia Ji[1], Chong Geng[1*]

[1] Department of Breast and Thyroid Surgery, Shandong Provincial Hospital Affiliated to Shandong First Medical University, Jinan, China

[2] Shandong Academy of Agricultural Science, Jinan, China

[3]Department of endocrine and metabolic diseases, Affiliated hospital of Wei Fang Medical University, Weifang, China

[4] Shandong Normal University, Jinan, China

***Corresponding to:**

Chong Geng, A13678826291@outlook.com

†These authors have contributed equally to this work and share first authorship

Keywords: Linoleic Acid, Alpha-Linolenic Acid, Colitis mouse model, Inflammation, Gut microbiota, 16S rRNA gene sequencing.

Table S1 Composition in g/kg of animal diets

Composition	Diet (g/1000 g)					
	LF-L	LF-M	LF-H	HF-L	HF-M	HF-H
Casein	189.58	189.58	189.58	233.06	233.06	233.06
L-Cystine	2.84	2.84	2.84	3.50	3.50	3.50
Cornstarch	298.59	298.59	298.59	84.83	84.83	84.83
Maltodextrin	33.18	33.18	33.18	116.53	116.53	116.53
Sucrose	331.77	331.77	331.77	201.36	201.36	201.36
Cellulose	47.40	47.40	47.40	58.26	58.26	58.26
Mixed Plant Oils	42.66 (LA/ALA, 1:1)	42.66 (LA/ALA, 5:1)	42.66 (LA/ALA, 50:1)	235.97 (LA/ALA, 1:1)	235.97 (LA/ALA, 5:1)	235.97 (LA/ALA, 50:1)
Mineral salt mix*	9.48	9.48	9.48	11.65	11.65	11.65
Calcium hydrogen phosphate	12.32	12.32	12.32	15.15	15.15	15.15
Calcium carbonate	5.21	5.21	5.21	6.41	6.41	6.41
Potassium citrate	15.64	15.64	15.64	19.23	19.23	19.23
Vitamin mix*	9.48	9.48	9.48	11.56	11.56	11.56
Bitartrate Choline	1.90	1.90	1.90	2.33	2.33	2.33

*Mineral salt mix and vitamin mix composition are according to Research diets S11026 and Research diets V10001.

Table S2 Compositions of blended plant oils with different LA/ALA ratios.

Fatty acid (mole %)	LA/ALA ratio		
	1:1	5:1	50：1
C8:0	-	-	-
C10:0	-	-	-
C12:0	-	-	
C14:0	0.55	0.03	0.03
C14:1	-	-	-
C15:0	-	-	-
C16:0	12.83	11.95	11.36
C16:1	0.33	0.06	0.05
C17:0	0.05	0.06	0.06
C17:1	0.0	0.03	0.03
C18:0	3.30	3.64	3.63
C18:1n9	37.31	36.51	35.43
C18:2n6	20.04	32.70	36.22
C18:3n3	21.28	6.13	0.69
C20:0	0.50	0.46	1.58
C20:4n6	0.01	-	-
C20:5n3	-	-	
C22:6n3	-	-	-
ΣSFA	17.24	17.15	16.68
ΣMUFA	37.75	36.65	37.57
ΣPUFA	36.96	37.83	36.92
SFA: MUFA: PUFA	1:2.19:2.14	1:2.20:2.32	1:2.25:2.21
Σ(n-6) fatty acids	18.63	34.71	36.23
Σ(n-3) fatty acids	18.33	6.13	0.70
n-6/n-3 ratio	1.02	5.66	51.76

Table S3 DAI Disease Activity Index Assessment Standards

Score	Body Weight	Stool characteristic	Rectal bleeding
0	No Body weight loss or body weight loss less than 1%	Normal stool consistency	negative hemoccult
1	Body weight loss between 1% and 5%	Soft stools (still formed)	positive hemoccult
2	Body weight loss between 5% and 10%	Very soft stools (wet)	Blood traces in stool visible
3	Body weight loss between 10 and 15%	Watery stools (Watery diarrhea)	Visible rectal bleeding
4	Body weight loss over 15%	---	---

Table S4 Histological Scores of Colon Damage

Score	Inflammation severity	Inflammation extent	Crypt damage
0	None	None	None
1	Mild	Mucosa	Basal 1/3 damaged
2	Moderate	Mucosa and submucosa	Basal 2/3 damaged
3	Severe	Transmural	Crypts lost, surface epithelium present
4	-	-	Crypts and surface epithelium lost
